# Neutrophilic proteolysis in the cystic fibrosis lung correlates with a pathogenic microbiome

**DOI:** 10.1186/s40168-019-0636-3

**Published:** 2019-02-13

**Authors:** Robert A. Quinn, Sandeep Adem, Robert H. Mills, William Comstock, Lindsay DeRight Goldasich, Gregory Humphrey, Alexander A. Aksenov, Alexei V. Melnik, Ricardo da Silva, Gail Ackermann, Nuno Bandeira, David J. Gonzalez, Doug Conrad, Anthony J. O’Donoghue, Rob Knight, Pieter C. Dorrestein

**Affiliations:** 1 Skaggs School of Pharmacy and Pharmaceutical Sciences, University of California San Diego, La Jolla, CA USA; 2 Center for Microbiome Innovation, University of California San Diego, La Jolla, CA USA; 3 Department of Biochemistry and Molecular Biology, Michigan State University, East Lansing, 48823 MI USA; 4 Department of Pediatrics, University of California San Diego, La Jolla, CA USA; 5 Department of Computer Science and Engineering, University of California San Diego, La Jolla, CA USA; 6 Department of Pharmacology, University of California San Diego, La Jolla, CA USA; 7 Department of Medicine, University of California San Diego, La Jolla, CA USA

## Abstract

**Background:**

Studies of the cystic fibrosis (CF) lung microbiome have consistently shown that lung function decline is associated with decreased microbial diversity due to the dominance of opportunistic pathogens. However, how this phenomenon is reflected in the metabolites and chemical environment of lung secretions remains poorly understood.

**Methods:**

Here we investigated the microbial and molecular composition of CF sputum samples using 16S rRNA gene amplicon sequencing and untargeted tandem mass spectrometry to determine their interrelationships and associations with clinical measures of disease severity.

**Results:**

The CF metabolome was found to exist in two states: one from patients with more severe disease that had higher molecular diversity and more *Pseudomonas aeruginosa* and the other from patients with better lung function having lower metabolite diversity and fewer pathogenic bacteria. The two molecular states were differentiated by the abundance and diversity of peptides and amino acids. Patients with severe disease and more pathogenic bacteria had higher levels of peptides. Analysis of the carboxyl terminal residues of these peptides indicated that neutrophil elastase and cathepsin G were responsible for their generation, and accordingly, these patients had higher levels of proteolytic activity from these enzymes in their sputum. The CF pathogen *Pseudomonas aeruginosa* was correlated with the abundance of amino acids and is known to primarily feed on them in the lung.

**Conclusions:**

In cases of severe CF lung disease, proteolysis by host enzymes creates an amino acid-rich environment that *P. aeruginosa* comes to dominate, which may contribute to the pathogen’s persistence by providing its preferred carbon source.

**Electronic supplementary material:**

The online version of this article (10.1186/s40168-019-0636-3) contains supplementary material, which is available to authorized users.

## Introduction

In the chronically infected cystic fibrosis (CF) lung, there is a severe microbial dysbiosis, where the organ becomes inundated with infectious agents, including bacteria, fungi, and viruses [1–3]. In response to this complex polymicrobial infection, the lung recruits high levels of neutrophils over decades, creating a highly inflammatory environment [1, 4]. The microbial composition of this community has been well characterized. It has been repeatedly shown that as the disease progresses and patients age, the community diversity decreases and pathogens, particularly *Pseudomonas aeruginosa*, come to dominate [1, 5, 6].

The chemical composition of this polymicrobial and hyperinflammatory lung environment has also been studied. Mucin, DNA, and amino acids are major constituents of CF sputum [7–10], and there is a high load of cellular and inflammatory lipids [11–13]. High amounts of antibiotics from both acute and chronic therapies [11], and microbial fermentation products including ethanol, acetate, 2-propanol, and 2,3-butanediol, are also found in airway secretions [14, 15]. Despite this well established knowledge of the CF microbial composition and growing understanding of the CF lung chemical environment, how the microbiome, metabolome and hyperinflammation collectively contribute to disease progression remains elusive.

Here we combine microbiome sequencing, metabolomics and peptidomics on adult CF sputum to analyze the relationship between microbial/chemical composition and disease severity. We use these results to propose a model of how extensive neutrophilic proteolysis in the lung generates abundant peptides and amino acids that promote the growth and persistence of pathogens, leading to more severe lung disease.

## Methods

### Sample collection

Sputum samples (*n* = 103) were collected from 88 adult CF patients (> 18 years) according to a UC San Diego institutional review board approved protocol for human subject research (#160078) (Additional file 1: Table S1) from the UC San Diego adult CF clinic during routine visits. The patients were selected based on positive diagnosis of CF with either genetic testing for mutations in both copies of the *CFTR* gene, positive sweat chloride test, or both. Patients having received a lung transplant were removed from the study. The clinical state of the patient was defined as either (a) “exacerbation” (clinical decision to treat with intravenous antibiotics at the time of sample collection), (b) on “treatment” (sample collected during intravenous antibiotic treatment), (c) “post treatment” (within 3 days after intravenous therapy was finished) or (d) “stable” (no inpatient treatment or exacerbation present; Additional file 1: Table S1). Patients first rinsed their oral cavity with a sterile solution of 7% saline, and a subset of mouth rinse samples were collected for analysis (*n* = 24; Additional file 1: Table S1). Induced sputum samples were then collected via inhalation of 7% hypertonic saline and expectoration of sputum into a sputum cup for a maximum of 30 min. The sample was homogenized with a 3-ml syringe without the needle and aliquoted in 1–2 ml volumes into cryovials. Both the sputum and mouth rinse cryovials were then immediately frozen in a dry liquid nitrogen dewar and stored at − 80 °C until analysis.

### LC-MS/MS

Sputum and mouth rinse samples were thawed, and two 100-μl aliquots of sample were added to separate Axygen® 1.5-ml microfuge tubes. There were two separate extractions done on these samples: an ethyl acetate/methanol procedure that was used for all statistical analyses and a 100% ethanol procedure to detect more polar amino acids after preliminary analysis. The primary extraction was done according to the methods described in [11]. The second involved the addition of 200 μL of HPLC grade ethanol to the samples and incubation at room temperature for 2 h. The samples were centrifuged at 10,000×*g* for 30 s, and the ethanol layer was removed for LC-MS/MS analysis. Blank samples comprised those that went through the entire extraction without any sample added to the microfuge tube.

The metabolite extracts were diluted fourfold in methanol containing an internal standard of 2 μM ampicillin for quality control (Thermo Fisher Scientific, Waltham, MA, USA) prior to LC-MS/MS. Chromatography was done using a Thermo Scientific™ UltraMate 3000 Dionex UPLC system (Thermo Fisher Scientific, Waltham, MA USA) followed by eluent analysis on a Bruker Daltonics® maXis™ impact™ qTOF mass spectrometer (Bruker, Billerica, MA, USA). Metabolites were separated using a Kinetex 1.7 μm C18 (50 × 2.10 mm) UPLC column. Mobile phases comprised of 98:2 and 2:98 ratios of water and acetonitrile, respectively, containing 0.1% formic acid were used and a linear gradient from 0 to 100% for a total run time of 840 s at a flow rate of 0.5 mL min^−1^ was used for separation.

### GC-MS

Samples with sufficient volume remaining to enable analysis (*n* = 91) were analyzed with GC-MS to assess the relationship between volatile metabolites and the microbial profiles. A 100-μL volume of sputum was aliquoted into 2-ml borosilicate vials and capped with a screw cap with silicon septum and stored at − 20 °C. Volatiles from the sample were extracted from the headspace using polydimethylsiloxane/divinylbenzene (PDMS/DVB) d_f_ 65-μm solid phase microextraction (SPME) fiber for 10 min at 160 °C. The fiber was inserted into the injector equipped with Merlin septum heated to 250 °C, and the adsorbed compounds were desorbed for 1 min. Quality controls of natural mint oil extract were run along with samples throughout the analysis to monitor instrument performance and SPME wear.

### Mass spectrometry data processing and analysis

Bruker .d files from the Maxis qTOF were lockmass-calibrated and converted to the .mzXML format using the Bruker Compass batch method processor in the DataAnalysis® software. Both the peak list and raw data were then uploaded to the MassIVE server and GNPS (http://gnps.ucsd.edu [16]) and made publicly available under accession ID MSV000080655. Molecular networks were built for annotation of metabolites and to visualize the data on GNPS. Annotations were obtained by matching spectra against the public GNPS MS/MS spectral library and a commercial library (MS/MS NIST17). The spectral annotation false discovery rate was calculated according to [17]. Feature finding for the LC-MS/MS data was performed using the MZmine 2 software [18]. The feature table with the area under the curve abundance of a corresponding MS^2^ node cluster IDs in GNPS was then output as a .txt file for statistical analysis. This enabled the linking of annotated metabolites through GNPS library searching with actual feature abundances from MS^1^.

The GC-MS data were processed with MZmine 2 [18] using the ADAP algorithm [19] (version ADAP-in-MZmine2.23, https://github.com/du-lab/ADAP-in-MZmine2). Data were uploaded to GNPS and searched against NIST 2017 and WILEY spectral libraries. The GC-MS data are available under MassIVE ID MSV000081150. Further description of mass spectrometry data processing is available in Additional file 2: Supplementary methods.

### DNA extraction and 16S rDNA amplicon sequencing

DNA extraction, 16S rRNA gene variable region 4 (V4) PCR, and amplicon preparation for sequencing were performed according to protocols benchmarked for the Earth Microbiome Project (EMP) found here: http://www.earthmicrobiome.org/protocols-and-standards/. Briefly, genomic DNA was extracted using the MagAttract DNA isolation kit (Qiagen, Carlsbad, CA), and the V4 region was PCR amplified in triplicate from each sample and combined.

### Microbiome data processing

The microbiome data was processed through the Qiita software (qiita.ucsd.edu). The data was demultiplexed and then rarified at a sequence sampling depth of 1000 reads before processing using the closed reference operational taxonomic unit (OTU) picking method. The resultant .biom files were used for downstream analysis.

### Peptidomics

The LC-MS/MS .mzXML files were loaded into PEAKS Studio 8.5 software [20] for de novo identification and searching against the UniProt human protein database. Label-free quantification was run through PEAKS Studio 8.5 [20]. A 1% false discovery rate (FDR) cutoff was used to integrate peaks with a 20 ppm mass error tolerance and a 6-min retention time window. Peptides were searched against the UniProt human protein database for identification. Quantification was normalized to the abundance of the total ion chromatograph. The human proteomics data was also validated with the MS-GF+ workflow through the identification of the same proteins after searching the human proteome [21].

### NE and cathepsin G (CG) assays

Once the MS and 16S rRNA gene amplicon sequencing was complete, all sputum samples with > 50-μL volume remaining (*n* = 89) were diluted 20-fold in Dulbecco’s phosphate buffer saline (D-PBS) and stored at − 20 °C. Samples were then diluted tenfold in D-PBS containing 0.01% Tween-20 and 50 μM Succinyl-Ala-Ala-Pro-Phe-aminomethylcoumarin (Bachem) and assayed for 1 h at room temperature in triplicate wells of a black round bottom 96-well plate. Hydrolysis of the fluorescent substrate was monitored in a Synergy HTX multi-mode reader (Biotek) using excitation and emission of 360 and 460 nm, respectively, and activity was expressed as a change in fluorescent units per second and normalized to the activity of 250 nM of human neutrophil CG (EMD Millipore). Samples were also diluted 1000-fold in D-PBS containing 0.01% Tween-20 and 50 μM methoxy succinyl-Ala-Ala-Pro-Val-aminomethylcoumarin (Alfa Aesar) and assayed as outlined above, except 5 nM of human neutrophil elastase (NE) (Athens Research) was used as a control enzyme.

### Culture experiments

A CF isolate of *P. aeruginosa* (VVP006) was used to test for protease activity against the NE substrate and for growth with and without added amino acids. For the protease assay, the strain was first grown on Todd Hewitt Agar, then inoculated into artificial sputum medium (ASM, recipe from [22]) and incubated at 37 °C for 48 h. The cultures were then pelleted in a benchtop centrifuge at 10,000×*g* for 30 s, and the supernatant diluted 20-fold in D-PBS containing 0.01% Tween-20 and 50 μM methoxy succinyl-Ala-Ala-Pro-Val-aminomethylcoumarin. Activity assays were performed in triplicate as outlined above. *P. aeruginosa* VVP006 was also grown in ASM with and without the amino acid components of the media, and optical density was measured after 48 h at 37 °C.

### Statistical analysis

Alpha diversity of the microbiome data was calculated using the Shannon index of the OTU table and on the metabolome data from the deconvoluted and deisotoped MS^1^ feature table after removal of background contaminants. Differences in alpha-diversity were tested using the Student *t* test. Beta-diversity of the mass spectrometry data was calculated using the Bray-Curtis dissimilarity and visualized with principal coordinate analysis (PCoA) through our in-house ClusterApp software (an interface for the R statistical software). Hierarchical clustering and silhouette plots were used to group the samples optimizing within group cohesion and between group separations. The reproducibility of the clustering was tested with jackknifing inter-quartile ranges visualized in three-dimensional PCoA space, for different rarefaction values (100, 1000, 1,000,000). Additionally, the groups detected by unsupervised clustering were subjected to permutation analysis of the multivariate data variance (PERMANOVA) using 999 permutations. Beta-diversity of the microbiome data was calculated using the weighted UniFrac distance [23] in the Qiita software (qiita.ucsd.edu), with hierarchical clustering and silhouette plots used to identify the clustering patterns in the PCoA. The original data used for this statistical analysis is available in this repository: https://github.com/DorresteinLaboratory/XSectionalCF, and the code for the statistical analysis is available here: https://github.com/DorresteinLaboratory/XSectionalCF/blob/master/XSectional.ipynb

The microbiome data was analyzed in the context of the two metabolome clusters by quantifying the relative abundance of each OTU per sample in each cluster and tested for significance with the Mann-Whitney *U* test. The microbiome data was further classified at the genus level as belonging to “pathogens” or “anaerobes” according to the Additional file 2: Supplementary methods.

Statistical differences between clinical and demographic data were calculated in relation to the two metabolome clusters using the Mann-Whitney *U* test. To ensure sample comparisons were completely independent, the same statistical test was done after removal of samples collected from the same patient and only tested on one sample from each patient (*n* = 88, keeping most recent sample from each patient; Additional file 3: Table S6). A random forests regression model was used to determine the association between the metabolomic and microbiome data and the target FEV1% from the same samples. The model was run in the R statistical software using the randomForest package with 5000 trees on the entire OTU dataset and the MS^1^ features. Correlations between the machine-learning projected FEV1% based on the random forests model and the actual measurement were tested using the Spearman correlation, and the variance explained by either the microbiome or metabolomic data was reported. Correlations between individual microbiome OTUs and target FEV1% were calculated using Spearman rank correlation and corrected for multiple comparisons using the Benjamini-Hochberg procedure. The OTU table was processed to include only those with a minimum abundance 0.001% of the entire dataset, leaving 516 OTUs.

Specific feature differences between the two metabolome clusters were identified using a supervised random forests classification in the R-statistical software package randomForest. The variable importance plot of this classification was then used to identify the differentially abundant metabolites in the dataset, which were visualized in the molecular networks. Significance between the variables of importance was calculated with the Mann-Whitney *U* test.

The activity of NE and CG and the relative abundance of HNP1 were tested in the two clusters using the Mann-Whitney *U* test for both the entire dataset and completely independent subset (Additional file 3: Table S6).

### *P. aeruginosa* and metabolite correlations

The abundances of selected metabolites known to be produced by the *P. aeruginosa* (the quinolones (HHQ and NHQ), a rhamnolipid (Rha-Rha-C10-C10), and pyochelin) were regressed against the normalized abundance of *Pseudomonas* OTU 4454529 in the same samples using the Pearson correlation (*r*). In addition, Pearson’s *r* was calculated on the metabolite-metabolite correlations based on their normalized abundances.

## Results

### Microbiome and metabolome alpha and beta diversity

**Fig. 1:**
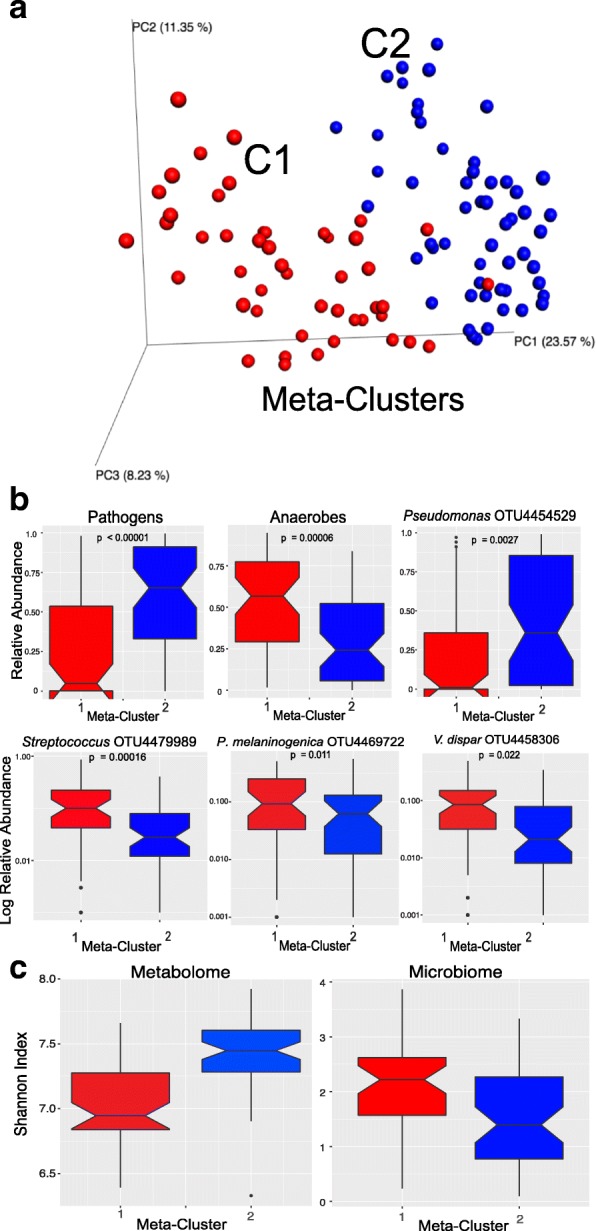
**a** PCoA plot of metabolomic data with the two hierarchical clusters highlighted (C1, C2). **b** Notch plots of the relative abundance of “pathogens,” “anaerobes,” and OTUs of interest in samples representing the two metabolome clusters. **c** Shannon index of metabolome and microbiome data in the two metabolome clusters

Silhouette plots, hierarchical clustering, and principal coordinate analysis (PCoA) with jackknifing inter-quartile ranges were used to visualize the beta diversity of the microbiome and metabolome data. Jackknifing and PERMANOVA were used to determine the significance of any clusters identified (Additional file 4: Figure S1). The metabolomic data were separated into two clusters consisting of 44 patients and 56 patients, which were robust to the jackknifing and PERMANOVA testing (Fig. 1, Additional file 4: Figure S1) (these metabolome clusters are hereafter referred to as meta-cluster 1 and meta-cluster 2, respectively). The microbiome profiles of the same samples belonging to these two metabolome clusters were also different. The microbiome profile of samples from meta-cluster 1 was enriched in anaerobic bacteria such as *Streptoccoccus* sp., *Prevotella melaninogenica*, and *Veillonella dispar*, whereas meta-cluster 2 was enriched in *P. aeruginosa* (Fig. 1).

The silhouette plot from hierarchical clustering of the sputum 16S rRNA gene sequencing data did not show strong clustering (Additional file 4: Figure S2). The microbiome data was instead driven by the abundance of the pathogens *P. aeruginosa* and *S. maltophilia* (Additional file 4: Figure S2). The PCoA plot showed a tight grouping of samples with a similar dominance of *P. aeruginosa*, while the remaining samples contained a more diverse community of anaerobic bacteria mixed with varying amounts of *P. aeruginosa* (Additional file 4: Figure S1).

There was an inverse relationship between the Shannon diversity of the microbiome and metabolome data in the two metabolomic clusters. Meta-cluster 1 had a significantly higher microbial diversity than meta-cluster 2 (Student’s *t* test, *p* < 0.0001), but the molecular diversity was significantly higher in meta-cluster 2 than meta-cluster 1 (Student’s *t* test *p* < 0.0001; Fig. 1). Regression analysis verified the negative relationship between microbiome and metabolome alpha diversity (Pearson’s *r* = − 0.345, *p* = 0.00062; Additional file 4: Figure S3). This discrepancy is due to the high abundance and diversity of peptides in meta-cluster 2 as described in detail below.

### Clinical state and multi-omics

**Fig. 2:**
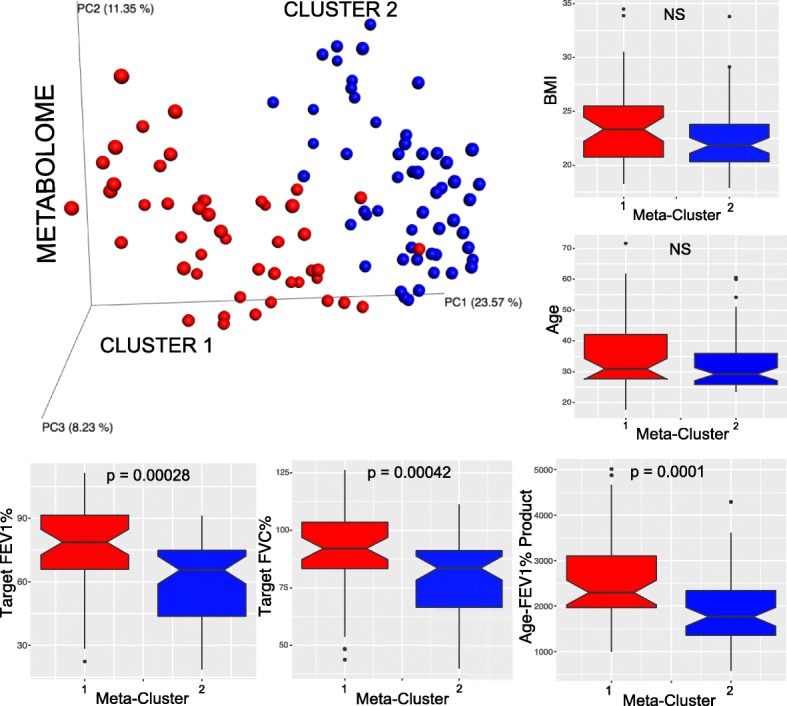
PCoA plot of the two metabolomic clusters and notch plots corresponding to the clinical data measures for target FEV1%, target FVC%, age-FEV1% product, age, and BMI within each cluster. The reported *p* value is from the Mann-Whitney *U* test. NS = not significant

The metabolomic data had strong signatures related to patient disease severity (target forced vital capacity (FVC), target forced expiratory volume in 1 s as percent of normal (target FEV1%), and the age-target FEV1% product [24]). All measures of lung function were significantly lower in patients belonging to meta-cluster 2 (Mann-Whitney *U* test, *p* < 0.05; Fig. 2). The demographic measures of these patients however (Additional file 1: Table S1) were not significantly different between the two metabolome clusters (age, weight, height, male/female, and on/off chronically inhaled or intravenous antibiotic therapy (chi-squared test)), except that BMI reached significance when tested with the Mann-Whitney *U* test on the independent dataset only (Additional file 3: Table S6). A random forests regression model of the metabolomic data and target FEV1% (i.e., highest FEV1% in previous 52 weeks) demonstrated that 19% of the data variance was explained by this measure of lung function and the correlation between the predicted and measured values was statistically significant (Additional file 4: Figure S4). Spearman correlations between molecular features and target FEV1% were calculated, and 382 molecular features were significantly negatively correlated with lung function after FDR correction (*p* < 0.01; Additional file 5: Table S2). Known metabolites within the 382 features included the amino acids, tryptophan (*rho* = − 0.401, FDR-corrected *p* = 0.0039), phenylalanine (*rho* = − 0.351, FDR-corrected *p* = 0.0080), and the dipeptide Ile/Leu-Pro (*rho* = − 0.356, FDR-corrected *p* = 0.0074) (Additional file 4: Figure S4). The isomers Ile and Leu could not be distinguished using this mass spectrometry approach.

The four microbiome clusters (micro-cluster 1–4) were not significantly different using a one-way ANOVA by any measure of disease severity or demographics. A random forests regression model was run on the complete microbiome data to determine if there was a relationship with target FEV1% in the same samples. The random forests did not identify a significant relationship between lung function and the microbiome profiles (Additional file 4: Figure S4). The relative abundance of the *P. aeruginosa* OTU did have a negative correlation with lung function, but this was not significant (Pearson’s *r* = − 0.225, FDR-corrected *p* > 0.05, target FEV1%). No other bacterial OTUs were significantly correlated with target FEV1% after the false discovery rate correction.

### Molecular differences between the metabolome clusters

**Fig. 3:**
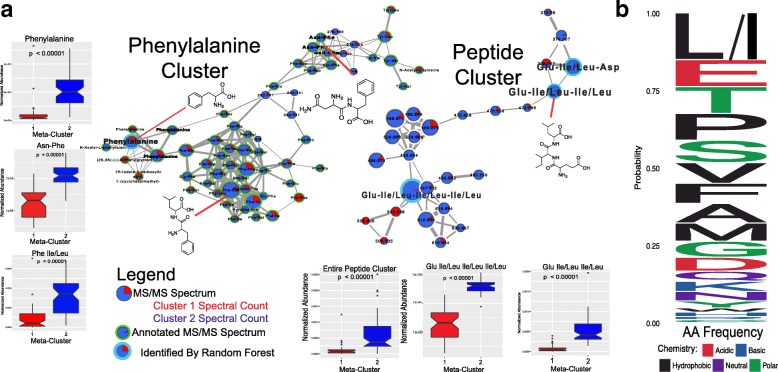
**a** Molecular networks of peptides and amino acids that strongly separated the two metabolomic clusters. Each node represents a consensus MS/MS spectrum and edges between the nodes represent spectral similarity as determined by the cosine score. The pie chart inside the nodes shows the number of spectra found in each cluster. Nodes with blue a green outline were annotated from GNPS MS/MS library searching and those with a blue outline were identified in the random forest variable importance plot. Notch plots of the MS^1^ normalized abundance of nodes of interest as well as the entire peptide networks are shown and the Mann-Whitney *U* test was used to determine significance. **b** Frequency rank plot of amino acid abundances in the de novo peptide sequencing data

To identify metabolites differentially abundant between meta-cluster 1 and meta-cluster 2, a random forests classification model was used with the samples classified by cluster membership. The variable importance plot (VIP) of this classification then identified which molecular features were most strongly contributing to the separation. MS/MS spectral matching against known library spectra in the GNPS database enabled putative annotation of these molecules. Our annotation rate in this dataset was 10.4% (771 nodes out of 7434; maximum FDR = 0.003). A metabolite with the sixth strongest classification was annotated as a tripeptide with the sequence Asp-Ile/Leu-Phe. Seven of the top 20 differential metabolites annotated through GNPS were connected to peptides in the molecular networks, including a putatively annotated peptide Glu-Ile/Leu-Ile/Leu-Ile/Leu, which was the second strongest classifier (Fig. 3). There were three large peptide molecular networks in the data that represented related di-, tri-, and tetra-peptides more abundant in meta-cluster 2. This indicated that the two metabolome clusters identified in the sputum samples were separated due to the contributions of various small peptides to the overall data. The abundance and diversity of these peptides contributed to the differences seen in overall Shannon diversity between the microbiome and metabolomic data. Furthermore, the free aromatic amino acids Phe and Trp were also observed to be differentially abundant between the two groups (Fig. 3, Additional file 4: Figure S5), although it must be noted that not all amino acids can be detected in our protocol due to insufficient retention on the C18 column used in this study. Antibiotics were also detected in the sputum metabolomic data; however, the relative abundance of these molecules in the two-metabolome clusters were not significantly different (Additional file 4: Figure S5).

### Peptidomics

Due to the abundant and diverse peptides in the LC-MS/MS data, we used peptidomics software for de novo sequencing and searched identified peptide spectra against the human proteome. The de novo sequencing allowed for assessment of beta-diversity and overall peptide abundance. A PCoA plot of the peptidomics data also identified two separate clusters of patient samples (Additional file 4: Figure S5). Patients belonging to peptide cluster 2 (*n* = 56) matched those belonging to meta-cluster 2 in the metabolomic data verifying that peptide abundance contributed to the initial group separation (56/56). In terms of abundance, meta-cluster 2 was found to have significantly more peptides (Mann-Whitney *U* test, *p* = 0.00036; Additional file 4: Figure S5). De novo sequencing also allowed for identification of the amino acid frequencies found in CF sputum peptides. The sequenced peptides were enriched in Ile/Leu, Glu, Phe, Tyr, Pro, Ser, Val, and Phe residues (Fig. 3).

Searching the human proteome identified 1079 unique peptides that matched to 89 different human proteins (1% FDR). The most abundant peptides were derived from E3 ubiquitin-protein ligase, calprotectin (S100-A9), the lipopolysaccharide-binding BPI-like 1 protein, histone-like *N*-methyltransferase SETD2, lactotransferrin, and a number of other housekeeping and neutrophil-associated proteins (Additional file 6: Table S3). Their abundance was tested for significance between the two metabolome clusters using the Mann-Whitney *U* test. Calprotectin (*p*< 0.0001), LMBR1-protein related to lipocalin (*p*< 0.0001), thymosin beta-4 (*p*< 0.0001), glyceraldehyde-3-phosphate dehydrogenase (*p*< 0.0001), neutrophil-gelatinase-associated lipocalin (*p*< 0.0001), lactotransferrin isoform 2 (*p*< 0.0001), E3 ubiquitin-protein ligase (*p*< 0.0001), bactericidal/permeability-increasing protein (*p*< 0.0001), and neutrophil elastase (*p* = 0.00058) were all significantly more abundant in the peptide-rich meta-cluster 2 coming from patients with more severe disease (Mann-Whitney *U* test; Additional file 4: Figure S6). Annotated human proteins were verified using the MS-GF+ peptidomics workflow, and the same most abundant proteins were detected [21] (Additional file 7: Table S4). Searching against the *P. aeruginosa* peptidome with MS-GF+ identified 97 unique proteins with a total of 100 peptide hits. In contrast, there were 964 human proteins identified representing 1697 unique peptides (Additional file 7: Table S4).

### Neutrophil enzyme activity and molecular relationships

**Fig. 4:**
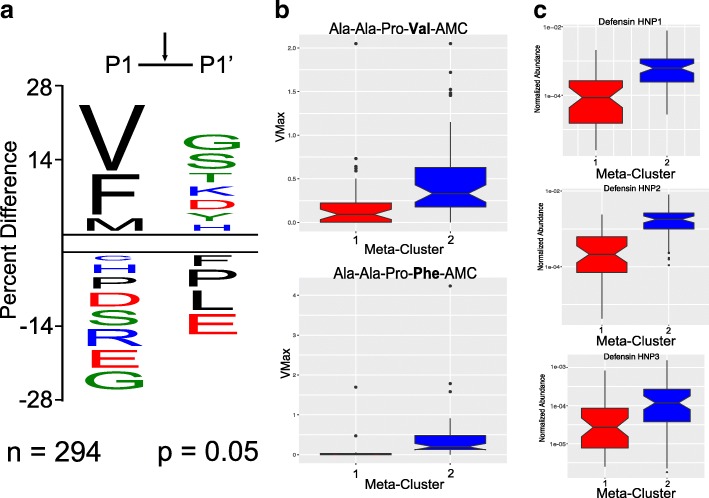
**a** Amino acid frequency plot of the P1 and P1′ sites as detected from peptide sequencing of LC-MS/MS data against the human genome. The size of the amino acid indicates the percent difference in abundance from others normalized to the relative abundance of each amino acid in the human peptidome. Those above the double line are significantly more abundant, while those below the line are significantly less abundant. Amino acids are colored based on their physiochemical properties (blue = positive charge, red = negative charge, black = hydrophobic, green = hydrophillic). **b** Protease activity in sputum samples using Ala-Ala-Pro-Val-AMC and Ala-Ala-Pro-Phe-AMC. The velocity of the reaction was measured as a change in fluorescent intensity per second. Activity from the two metabolomic clusters is shown as notch plots. **c** Notch plots of the normalized abundance of human neutrophil peptides in sputum samples from the two metabolomic clusters

We anticipated that proteases were responsible for production of peptides detected in the sputum samples and that the terminal amino acids would give insight into the enzymes responsible for the peptide cleavage activity. In protease nomenclature, cleavage of the scissile bond occurs between the P1 and P1′ amino acids. We generated a sequence motif by comparing the frequency of amino acids in the P1 position (found at C-terminus of cleaved products from the sputum peptidome) and the P1′ position (found at the N-terminus) to the frequency that these amino acids exist in the human proteome (Fig. 4A). Using a significance cut-off of 0.05, Val, Phe, and Met were enriched in the P1 position while Gly, Ser, Thr, Lys, Asp, Tyr, and His were found with high frequency in the P1′ position. Amino acids below the *x*-axis were rarely or never found in these positions. We assayed a subset of the sputum samples (*n* = 89) with synthetic substrates that have Val or Phe in the P1 position, directly adjacent to a cleavable fluorescent reporter molecule, 7-amino-4-methylcoumarin (AMC). Meta-cluster 2 was found to have significantly more activity than meta-cluster 1 for both substrates (Mann-Whitney U-test, *p* < 0.0001) (Fig. 4b). These data correlate the abundance of peptides found in the meta-cluster 2 sputum samples with an increased amount of neutrophil-mediated proteolysis. In addition, the antimicrobial neutrophil proteins HNP1, 2, and 3 detected in the LC-MS/MS data (Additional file 4: Figure S7) were also more abundant in cluster 2 (Mann-Whitney *U* test, p < 0.0001; Fig. 4).

To determine if the protease activity found in the sputum could have been due to an enzyme secreted by *P. aeruginosa*, the P1-Val fluorescent substrate was incubated with conditioned media from bacterial culture of *P. aeruginosa* CF isolate. No cleavage of the substrate was detected indicating that proteases in sputum were unlikely to be from this pathogen.

### *P. aeruginosa* and amino acid metabolism

The LC-MS/MS data showed that the metabolites tryptophan and phenylalanine were significantly correlated with the abundance of *P. aeruginosa* (Spearman *rho*: Trp = 0.397, *p* = 4.0 × 10^−5^, Phe = 0.391, *p* = 5.0 × 10^−5^). GC-MS data were used to further assess the relationships between the microbiome and primary metabolites in the sputum samples. The relative abundance of *P. aeruginosa* was regressed against known metabolites in the GC-MS data. The bacterium was positively correlated to indole (Pearson’s *r* = 0.431, *p* = 4.8 × 10^−5^, Spearman *rho* = 0.480, *p* < 0.001) and phenylacetic acid (Pearson’s *r* = 0.431, Spearman *rho* = 0.400, *p* = 0.00020; Additional file 4: Figure S8); both of which can be produced from amino acid breakdown. Our previous studies have shown that *P. aeruginosa* grows to a high density in ASM that is rich in amino acids and proteins [22]. When amino acids were omitted from this culture media, but protein remained, a non-mucoid *P. aeruginosa* CF isolate failed to grow (Additional file 4: Figure S9). This indicated that this strain (closely related to the Liverpool epidemic strain LES431) was unable to generate sufficient amino acids from enzymatic degradation of proteins in the ASM culture media and required free amino acids for growth. The strain used in this study was from a patient with a target FEV1% of 87.1%; thus, it may not represent the highly adapted strains from patients with more severe disease.

### *P. aeruginosa* specialized metabolite production in the two metabolomic states

**Fig. 5:**
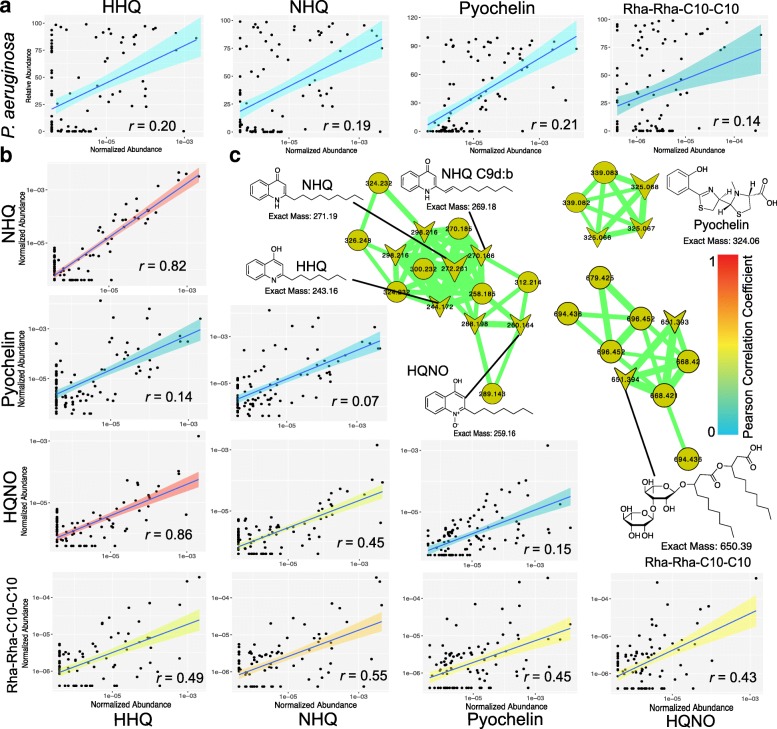
**a** Regression of *P. aeruginosa* relative abundance in 16S rRNA gene amplicon profiles and the levels of its metabolites in the same samples. **b** Regressions between the metabolites themselves. The linear regression line, its 90% CI, and the Pearson correlation *r* value are also shown. Regressions are shown pairwise in a matrix between each molecule. The 90% CI is colored according to the Pearson’s *r* to aid in visualization according to the color legend. **c** Molecular clusters of quinolones, rhamnolipid, and pyochelin and the structures corresponding to the metabolites compared in the regressions

Quinolones, rhamnolipids, pyochelin, and one phenazine were detected in the LC-MS/MS data (Fig. 5), and their abundance was compared in the two metabolome clusters. The quinolones 2-nonyl-4-hydroxy-quinolone (NHQ) (31/103 samples) and 2-heptyl-4-hydroxy-quinoline (HHQ) (32/103 samples) and the siderophore pyochelin (46/103 samples) were the most prevalent across the dataset and were therefore compared between the two meta-clusters. Levels of all three metabolites were higher in sputum samples from the Pseudomonas-dominated meta-cluster 2, although only pyochelin was statistically significant (Additional file 3: Table S6, Additional file 4: Figure S10). We thus compared the relative abundance of *P. aeruginosa* and its specialized metabolites in the same samples across both datasets. There was no correlation between the amount of *P. aeruginosa* and the abundance of its metabolites (Fig. 5). In many cases, *P. aeruginosa* was highly abundant in the microbiome profile, but no metabolites were detected. In contrast, *P. aeruginosa* metabolites were never detected in the absence of reads from the bacterium in the 16S rRNA gene profiles. The *P. aeruginosa* metabolites themselves, however, were all positively correlated with each other, particularly the various quinolones, with a strong linear relationship (Fig. 5).

## Discussion

This study found that the sputum metabolome of adult CF patients (> 18 years of age) existed in two states. One was associated with less severe disease, low chemical diversity, and high microbial diversity, while the other state was found in patients with more severe disease, higher chemical diversity, and lower microbial diversity, where pathogens such as *P. aeruginosa* were dominant. The existence of these two molecular states reflects the conditions of the lung in these two patient populations and can explain aspects of CF disease progression. In lungs with more severe disease, rampant protease activity resulted in a unique metabolomic signature that consisted of abundant and diverse degraded proteins products and amino acids, driving the clustering of patients into one of two molecular states.

The metabolomic data reflected lower lung function more strongly than the microbiome data. In the literature, a relationship between decreasing microbial diversity and disease progression is well-supported [1, 5]. Here, we also found signatures of declining lung function in the metabolomic data, and many molecules were negatively correlated with target FEV1%, particularly phenylalanine and tryptophan. Thus, monitoring of the metabolome for peptides and amino acids may aid in identifying patients whose disease is progressing to a more severe state. Furthermore, there is great potential to identify biomarkers of lung function decline in metabolomic data that can be supported with microbiome profiling and measures of inflammatory load.

The two molecular states were separated by a differential abundance of amino acids and peptides. Peptides frequently contained Val, Phe, and Met at the carboxyl termini, which are situated in the P1 position of the protease active site just prior to cleavage. In addition, the amino termini of these peptides were enriched with small residues such as Gly and Ser situated in the P1′ position. This cleavage pattern was described previously by Schilling and Overall [25] when human neutrophil elastase (NE) and cathepsin G (CG) were incubated with a cellular extract from human embryonic kidney cells. NE had a preference for cleavage between Val-Ser and Val-Gly bonds while CG had a preference for Phe-Gly, Phe-Ser, Met-Gly, and Met-Ser. These assays indicated that the peptides detected in the mass spectrometry data were being produced by these neutrophil proteases. Accordingly, neutrophil proteins (including NE itself) were found to be more abundant in the metabolome of patients with reduced lung function enriched in peptides. We therefore anticipated that there would be a concomitant increase in protease activity in these same patient samples, and our fluorescent reporter substrates containing P1-Val and P1-Phe confirmed this, showing abundant NE and CG activity in sputum from patients with reduced lung function. NE has been shown to be highly active in CF lungs and associated with lung function decline [26, 27]. Here, we support this finding and also implicate a role for CG in patients with declining lung function. Analysis of the peptide sequences indicated that the action of these enzymes was primarily on host proteins, including those related to basic cellular metabolism, but particularly those related to a neutrophilic inflammatory response. This is further supported by the corresponding high abundance of host α-defensins, also sourced from neutrophil granules [28], and neutrophil peptides, particularly calprotectin*.* Few *P. aeruginosa* peptides were discovered in the data, even in patients whose lungs were dominated by this bacterium, indicating that the vast majority of peptides detected were derived from the host. *P. aeruginosa* also produces proteases that may contribute to lung proteolysis [29]; however, conditioned media from this bacterium did not have enzymes that could cleave the fluorescent NE substrate. In summary, our data shows that in patients with more severe lung disease, neutrophil proteases degrade host proteins into peptides and amino acids and these metabolites can be found in CF lung mucus.

Several studies have shown that the *P. aeruginosa* preferentially feeds on amino acids in the CF lung [30–33]. It also upregulates genes for branched chain amino acid catabolism and represses those for their anabolism when grown in CF sputum [34]. This study supports these findings as the adult sputum metabolome contained abundant small peptides enriched with these amino acids (Ile/Leu and Val). Furthermore, patients with abundant peptides had higher levels of *Pseudomonas* in their microbiome, and its abundance was positively correlated with free amino acids and the by-products of their metabolism. The genomic evolution of *P. aeruginosa* also reflects the availability of these compounds as it becomes auxotrophic for certain amino acids during adaptation to the CF lung, particularly those with branched-chained residues and methionine [10, 35]. Thus, several lines of evidence from the literature [30–35], and from this study, indicate that there may be an important link between neutrophil protease activity and *P. aeruginosa* catabolism. The action of neutrophil proteases could contribute to shaping the niche space that *P. aeruginosa* comes to dominate in a severely diseased CF lung by providing its preferred carbon source.

The finding that microbial diversity and metabolite diversity have a negative relationship was an intriguing phenomenon that has been previously reported in sputum in a smaller study [11]. It would normally be expected that chemical diversity would increase with a concomitant increase in microbial diversity, yet this study showed the contrary. This discrepancy is due to the differential nature of the two omics datasets (Additional file 8). In 16S rRNA gene microbiome studies, PCR amplification targets a universal bacterial gene, providing information about the population and relative abundance of bacterial cells containing this gene. Metabolomics does not target a particular lineage of the tree of life; instead, extraction methods for mass spectrometry sample an untargeted pool of compounds in a complex sample. Thus, we propose that microbial and metabolite diversity in the CF lung did not correlate due to the greater neutrophilic inflammation in patients dominated by pathogens, such as *P. aeruginosa*, and the rampant proteolysis associated with that inflammation. Patients with a low microbial diversity have higher protease activity, which increases the extracted metabolite diversity via the detection of peptides. Further research into the relationship between microbial and metabolite diversity is warranted to better understand how microbes and host cells contribute to the nature of their surrounding chemical environment.

There was poor correlation between the relative abundance of *P. aeruginosa* in a sputum sample and the abundance of its specialized metabolites that are important for its virulence. Sputum samples with high relative abundance of *P. aeruginosa* often had low or undetectable amounts of quinolones, siderophores, and rhamnolipids from the bacterium. This demonstrates that there is an inconsistency between the detection of *P. aeruginosa* by 16S rRNA gene amplification and the detection of its metabolite production. Microbiome studies based on PCR amplification of 16S rRNA genes can amplify DNA from both live and dead cells [36]; the latter of which can persist for long periods [37]. The poor correlation between metabolite-based and DNA-based detection methods may therefore be due to the contrasting results from PCR amplification of DNA from metabolically inactive cells compared to detection of molecules produced during active metabolism. Treatment with dyes such as propidium monoazide should be further explored to determine the contribution of “dead DNA,” as it has been shown to change the abundance of *P. aeruginosa* [36]. On the other hand, many of the metabolites from *P. aeruginosa* were highly correlated to each other. This shows that production of these molecules was occurring concomitantly in the same sputum samples from within and across different metabolic pathways. Quinolones were highly correlated to each other and the rhamnolipid Rha-Rha-C10-C10, though a poor correlation was found with the siderophore pyochelin, possibly due to the longer half-life of this iron chelator. Thus, we propose that detection of *P. aeruginosa* metabolites, particularly HHQ and NHQ, may be informative for clinicians by demonstrating that a patient’s sample has active metabolism of the pathogen. As the use of multi-omics approaches in the microbiome field grows, further research into the relationship between microbial and metabolite abundance is needed.

In light of our results, we propose that there is a link between neutrophil proteolysis and the dominance of *P. aeruginosa* in cases of severe CF disease. We hypothesize that the CF lung becomes a favored environment for persistence of *P. aeruginosa* in part because this bacterium promotes recruitment of neutrophils to the lung and their proteases generate peptides and amino acids that are a favored carbon source of the pathogen [30–33, 38]. *P. aeruginosa*’s inherent resistance to neutrophilic attack [39], through its growth in biofilms and production of virulence factors [40–43], may explain its persistence and dominance in this amino acid rich environment. This proposed model creates a positive-feedback loop where an increased inflammatory load produces more amino acids promoting the expansion of *P. aeruginosa’s* growth and progressive lung function decline. Though this hypothesis was generated based on the multi-omics data presented here and we are unable to determine causality, this study expands on a larger model describing the microbial ecology in the CF lung called the *Climax and Attack Model* proposed by Conrad et al. [44, 45]. In addition, we provide further knowledge about the chemical composition of a severely diseased CF lung that is associated with the *Climax Community* of highly antibiotic resistant pathogens [44, 45]. Future work to understand the relationship between neutrophil proteolysis and amino acid metabolism by *P. aeruginosa* may shed light on its dominance in this highly inflamed environment. Our data also indicates that anti-NE and anti-CG treatments should be further investigated for their potential to reduce proteolysis in CF [46]. These drugs may not only reduce pulmonary inflammation, as has previously been shown [47], but may also inhibit the growth of *P. aeruginosa* by reducing the availability of its primary carbon source.

## Additional files

Additional file 1: **Table S1.** Samples and clinical information for patients included in this study. (XLSX 56 kb)

Additional file 2 ᅟSupplementary methods. Description of mass spectrometry data processing (DOCX 23 kb)

Additional file 3: **Table S6.** 16S rRNA gene read abundance for sputum samples and blanks after illumina sequencing. (XLSX 43 kb)

Additional file 4: Supplemental figures and legends. (DOCX 8840 kb)

Additional file 5: **Table S2.** Correlations between metabolites and 16S rRNA gene OTUs with Target FEV1%. (XLSX 438 kb)

Additional file 6: **Table S3.** Peptidomics data on human proteome as produced using PEAKS software. (XLSX 87 kb)

Additional file 7: **Table S4.** Human and *Pseudomonas aeruginosa* peptides from MS-GF+ proteomics search against human and *P. aeruginosa* SwissProt genomes. (XLSX 103 kb)

Additional file 8: **Table S5.** *p* values from complete dataset and independent dataset (1 sample each patient) for Mann-Whitney *U* tests of different variables in this study. (XLSX 9 kb)
