# Supplementary material for: Neutrophilic proteolysis in the cystic fibrosis lung correlates with a pathogenic microbiome
Source: Microbiome. 2019 Feb 13;7:23. doi: 10.1186/s40168-019-0636-3 (PMC6375204; doi:10.1186/s40168-019-0636-3)
Supplement: Supplementary file 2 — ᅟSupplementary methods. Description of mass spectrometry data processing (DOCX 23 kb) [file 40168_2019_636_MOESM2_ESM.docx]

**Neutrophilic Proteolysis in the Cystic Fibrosis Lung Shapes a Pathogenic Microbiome**

Quinn et al. Microbiome. Submission

Supplementary Methods

*Metabolite Extraction*

A 200 μl volume of LC-MS grade ethyl acetate was added to the sample, vortexed on max speed for 10 seconds and then extracted at room temperature for 2 hours. The mixture was spun at 10,000 x g for 30 s in a tabletop centrifuge and the ethyl acetate layer was removed to a fresh tube. A 200 μl volume of LC-MS grade methanol was added to the remaining sample and extracted at 4°C overnight. The centrifugation was repeated and the methanol layer was added to the lyophilized ethyl acetate extract.

*LC-MS/MS*

The mass spectrometer was tuned using Tuning Mix ES-TOF (Agilent Technologies) at a 3 mL min^-1^ flow rate once a day during the run. For accurate mass measurements, lock mass internal calibration used a wick saturated with hexakis (1H,1H,3H-tetrafluoropropoxy) phosphazene ions (Synquest Laboratories, *m/z* 922.0098) located within the source. Full scan MS spectra (*m/z* 50 – 2000) were acquired in the qTOF and the top ten most abundant ions in a particular scan were fragmented using collision induced dissociation at 35 eV for +1 ions and 25 eV for +2 ions in the collision cell. Data-dependent automatic exclusion was used such that an ion was fragmented upon its first detection, then fragmented twice more, but not again unless its intensity was 2.5x the previous fragmentation. This exclusion method was removed after 30 seconds and the mass spectrometer would repeat its ion detection and data-dependent automatic exclusion cycle.

*GC-MS Protocol.*

The GC protocol analysis included: cryofocusing on the head of the column at -10 °C for 1.25 min; 100 °C/min oven ramp to 40 °C (hold of 0.1 min), 15 °C/min oven ramp to 280 °C (hold of 0.1 min), ramp to 320 °C and a 3 min hold period to purge the column. The helium carrier gas was set to constant 2 mL/min flow and a splitless injection mode was applied. The scanned *m*/*z* range in a single quadrupole was 35-350 Da.

*Data Processing Parameters.*

The parameters of the GNPS molecular networks and library search were set as follow: precursor and fragment ion mass tolerance of 0.03, minimum cosine of 0.65 and a minimum fragment matched peaks of 3, MS-Cluster on. (GNPS network link: <https://gnps.ucsd.edu/ProteoSAFe/status.jsp?task=2e5b798bde194d5e92c9893af71319c3>)

The MZmine parameter of the workflow for LC-MS were as follows: MS^1^ noise level of 5000, MS^2^ noise level of 50; chromatograms were built with a minimum time span of 0.01, height of 10000 and tolerance of 20 ppm; chromatograms were deconvoluted with the base-line cutoff algorithm at a minimum peak height of 10,000 and baseline of 1000, a peak duration range of 0.01 to 1 min, and a scan pairing range of 0.03 Da; peaks were grouped to remove isotopes; chromatograms were aligned with a retention time tolerance of 0.2 min and a *m/z* tolerance of 0.03 Da. Metabolites detected in blanks and quality control standards were removed and the table was truncated to include only the 5000 most abundant features.

GC-MS parameters were set as follows: crop filter (0.35-25.0 min; m/z 20-350), noise level MS1 (7,000); ADAP chromatogram builder (min group size: 5; group intensity threshold 15,000; min highest intensity 10,000; *m*/*z* tolerance 0.7 Da); smoothing (filter width: 5); chromatogram deconvolution module (wavelets ADAP: S/N threshold: 0.5; peak width multiplicity: 1.0; abs (wavelet coeffs): true; min feature height: 7,000; coefficient area threshold 1.0; peak duration range: 0.005-0.7; RT wavelet range: 0.0001-0.04); ADAP decomposition module (Min cluster distance: 0.001 min; Min cluster size: 3; Min cluster intensity: 100; find shared peaks: false; Min edge-to-height ratio: 0.2; Min sharpness: 10.0; shape-similarity tolerance: 90; choice of Model Peak based Sharpness; ADAP aligner module (Min confidence:0.3; Retention time range 0.125 min; score tolerance 0.3; score weight: 0.3; EIC score: retention time); peak finder module (minimum absolute intensity of 0.05; retention time tolerance 1.5 min; and *m*/*z* tolerance of 0.45 Da).

*16S rRNA Gene PCR Protocol.*

The PCR mixtures contained 13 μl MoBio PCR water, 10 μl 5 Platinum Hot Start MasterMix (Life Technologies), 0.5 μl both the barcoded forward and reverse primers (515f and 806r; 10 μM final concentration), and 1.0 μl genomic DNA. Thermocycling consisted of ramping to 94°C for 3 min (denaturation), with amplification proceeding for 35 cycles at 94°C for 45 s, 50°C for 60 s, and 72°C for 90 s, followed by a final extension for 10 min at 72°C. After amplification, the DNA concentration was quantified using PicoGreen double-stranded DNA (dsDNA) reagent in 10 mM Tris buffer (pH 8.0). A composite sample for sequencing was created by combining equimolar ratios of amplicons from the individual samples, followed by ethanol precipitation to remove any remaining contaminants and PCR artifacts.

*Classification of OTUs as ‘Pathogens’ or ‘Anaerobes’.* Pathogens comprised OTUs from the genera: *Pseudomonas, Stenotrophomonas, Staphylococcus, Achromobacter* and *Burkholderia* and anaerobes comprised the genera: *Prevotella, Streptococcus, Veillonella, Fusobacterium, Gemella, Granulicatella, Leptotrichia, Actinomyces, Bulleidia, Oribacter* and *Atopobium*. These classifications were done similarly to those in [1,2] and reflect membership of the ‘*Climax’* and ‘*Attack’* communities proposed by Conrad et al. [3]. Pathogens represent those bacteria cultured routinely in clinical labs as ‘pathogens’ with ‘anaerobes’ representing the most abundant anaerobic genera found in CF microbiome profiles.

*Peptidomics Search Parameters.* *De novo* error tolerance parameters were used according to PEAKS default qTOF settings, 0.1 Da parent mass error tolerance, 0.1 Da fragment mass error tolerance. The search settings included no added restriction enzymes, variable dehydration, acetylation (N-Term), oxidation (M), and ubiquitination. The max variable post-translational modifications per peptide was set to 3. *De novo* sequence tags were first filtered to keep only those with an average local confidence above 50%. Only amino acids with 75% local confidence were used in reporting the N and C terminal residues.

The MS-GF+ search parameters were a parent mass tolerance set to 0.1 Da and spectrum level FDR set to 0.1. Identified peptides were considered only if the MS-GF+ E-value was less than 1 x 10^-10^. MS-GF+ was also used to identify and quantify *P. aeruginosa* peptides through searching against the PAO1 proteome with the same parameters.

1. Quinn RA, Whiteson K, Lim YW, Zhao J, Conrad D, Lipuma JJ, et al. Ecological networking of cystic fibrosis lung infections. npj Biofilms Microbiomes. 2016;2.

2. Carmody LA, Caverly LJ, Foster BK, Rogers MAM, Kalikin LM, Simon RH, et al. Fluctuations in airway bacterial communities associated with clinical states and disease stages in cystic fibrosis. Chotirmall SH, editor. PLoS One [Internet]. Public Library of Science; 2018 [cited 2018 Mar 13];13:e0194060. Available from: http://dx.plos.org/10.1371/journal.pone.0194060

3. Conrad D, Haynes M, Salamon P, Rainey PB, Youle M, Rohwer F. Cystic Fibrosis Therapy: A Community Ecology Perspective. Am J Respir Cell Mol Biol [Internet]. 2012 [cited 2013 Feb 1];48:150–6. Available from: http://www.ncbi.nlm.nih.gov/pubmed/23103995
