# Supplementary material for: Neutrophilic proteolysis in the cystic fibrosis lung correlates with a pathogenic microbiome
Source: Microbiome. 2019 Feb 13;7:23. doi: 10.1186/s40168-019-0636-3 (PMC6375204; doi:10.1186/s40168-019-0636-3)
Supplement: Supplementary file 4 — Supplemental figures and legends. (DOCX 8840 kb) [file 40168_2019_636_MOESM4_ESM.docx]

**Supplemental Figures Legends and Figures.**

**Figure S1.** Silhouette plot of hierarchical clusters identified in the CF sputum metabolome data and their variance after jackknifing the data. Results from PERMANOVA testing of the two clusters is shown.

**Figure S2.** A) Silhouette plot of hierarchical clusters identified in the CF sputum microbiome data. b) PCoA plot of the 16S rRNA amplicon sequencing data from the CF sputum samples with the 4 significant hierarchical clusters highlighted (C1-C4). c) Notch plots of the relative abundance of pathogens of interest in the four microbiome clusters.

**Figure S3.** Shannon diversity correlation of metabolome and microbiome data.

**Figure S4.** Results of random forests measured vs. predicted FEV1% based on the a) microbiome and b) metabolomic data. The random forests machine-learning algorithm uses the FEV1% from each sample to identify if there is a relationship with that linear value in the microbiome data patterns. The output of the regression model is a predicted target FEV1% based on the microbiome data, if the actual and predicted values do not coincide this indicates that the microbiome data did not strongly reflect the lung function measure. The Spearman’s *rho* and % of variance explained by the FEV1% variable is also shown. Regressions of the normalized abundance of tryptophan, phenylalanine and the peptide I/L-Pro identified in the metabolomic data with the FEV1%.

**Figure S5.** a) PCoA analysis of the de novo peptidomics data highlighting the two hierarchical clusters. b) Mean abundance of de novo sequenced peptides in patients from the two metabolome cluster. c) Mean abundance of NE mapping peptides in the two metabolome clusters. d) Tryptophan abundance in the two meta-clusters of patients. e) Normalized mean abundance of antibiotics detected in the metabolic data from patients representing the two meta-clusters. Trimethoprim, aztreonam, doxycycline, levofloxacin, sulfamethoxazole, ciprofloxacin and azithromycin are summed, and their distributions of abundance presented as notch plots.

**Figure S6.** Validation of peptidomics results Peaks software on LC-MS/MS data. a) Peptide mapping to BPIB1. b) MS^1^ profile of BPIB1 peptide m/z936.17. c) Peaks annotation of b and y ions and manual sequencing of same MS^2^ spectrum of BPIB1. d) Peptide mapping to calprotectin subunit S100-A8. e) MS^1^ features and GNPS molecular network of S100-A8. f) Peaks annotation of b and y ions and manual sequencing of same MS^2^ spectrum of S100-A8. g) MS^1^ profile and molecular network of de novo sequenced peptide *m/z*808.3724. h) Manual validation of de novo sequencing of *m/z*808.3724. i) MS^1^ profile and molecular network of de novo sequenced peptide *m/z*788.436. i) Manual validation of de novo sequencing of *m/z*788.436.

**Figure S7.** MS1 spectra and isotopic patterns of HNP1–3 in LC-MS/MS data.

**Figure S8.** Regression between *P. aeruginosa* relative abundance and normalized abundance of indole and phenylacetic acid.

**Figure S9.** Optical density of *P. aeruginosa* VVP006 growth in artificial sputum media with and without amino acids.

**Figure S10.** Normalized abundance of *P. aeruginosa* metabolites in the two metabolomic clusters. Figure S11 Number of samples in the sequencing dataset and the frequency of reads per sample. (DOCX 9057 kb)

Fig. S11. Number of samples in the sequencing dataset and the frequency of reads per sample.

Fig. S1

Fig. S2

Fig. S3

Fig. S4


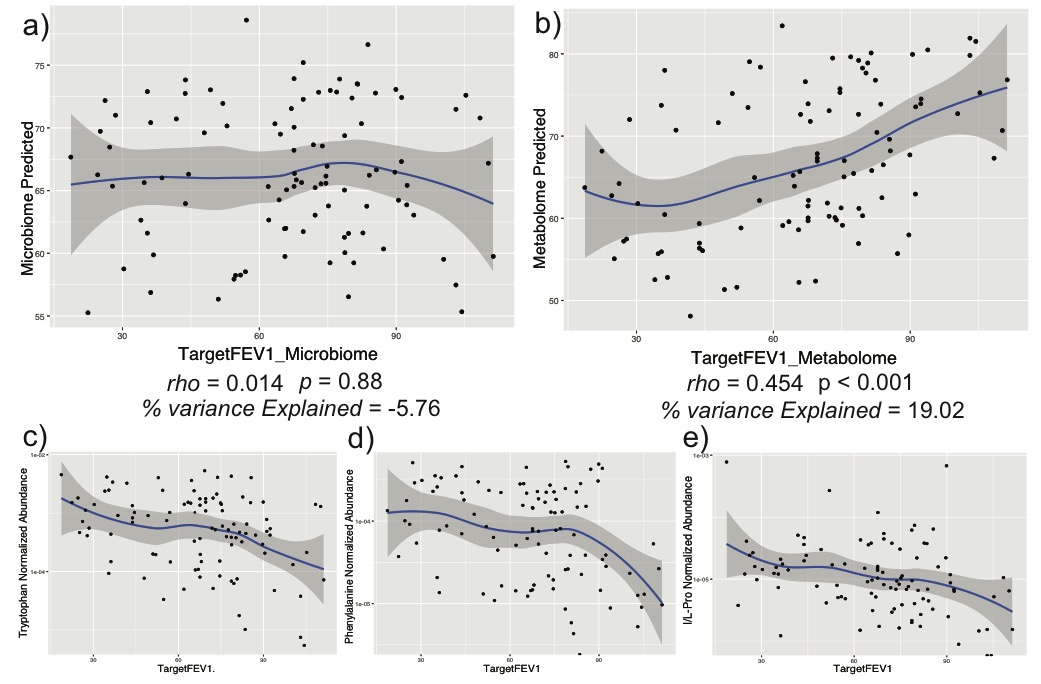


Fig. S5

Fig. S6

Fig. S7

Fig. S8

Fig. S9

Fig. S10

Fig. S11.
